# Unexpected Non-acid Drainage from Sulfidic Rock Waste

**DOI:** 10.1038/s41598-019-40357-4

**Published:** 2019-03-13

**Authors:** Andrea R. Gerson, Peter J. Rolley, Catherine Davis, Sandrin T. Feig, Stephen Doyle, Roger St. C. Smart

**Affiliations:** 1 Blue Minerals Consultancy, Wattle Grove, Tasmania, Australia; 2 Kanmantoo Copper Mine, Éclair Mine Road, Kanmantoo, South Australia Australia; 3 Central Science Laboratory, University of Tasmania, Hobart, Tasmania Australia; 4 Institute for Photon Science and Synchrotron Radiation (IPS), Karlsruhe Institute of Technology (KIT), Hermann-von-Helmholtz-Platz 1, 76344 Eggenstein-Leopoldshafen, Germany

## Abstract

Most rock extraction sites, including mine sites and building construction sites, require a plan to assess, and mitigate if present, the risk of acid mine drainage (AMD). AMD is typically the major environmental concern where sulfide minerals are present in the excavated material and AMD prediction and remediation is based on internationally-accepted acid-base accounting (ABA) tests of representative field samples. This paper demonstrates that standardized ABA tests may not always be provide the correct AMD classification for commonly occurring waste rocks containing low-pyrite and -carbonate due to mineralogic assumptions inherent in their design. The application of these standard ABA tests at a copper mine site in South Australia resulted in the classification of a portion of its waste material as potentially acid forming in apparent contradiction to long term field measurements. Full definition of the sulfide and silicate minerals enabled re-evaluation of the weathering reactions occurring. The overall rate of neutralisation due to silicate dissolution was found to always exceed the rate of acid generation, in agreement with field observations. Consequently, the waste rock was redefined as non-acid forming. The methods developed represent a significant advance in AMD prediction and more strategic, cost-effective environmental planning, with potential for reclassification of wastes with similar characteristics.

## Introduction

### The Hillgrove Mine

**Figure 1:**
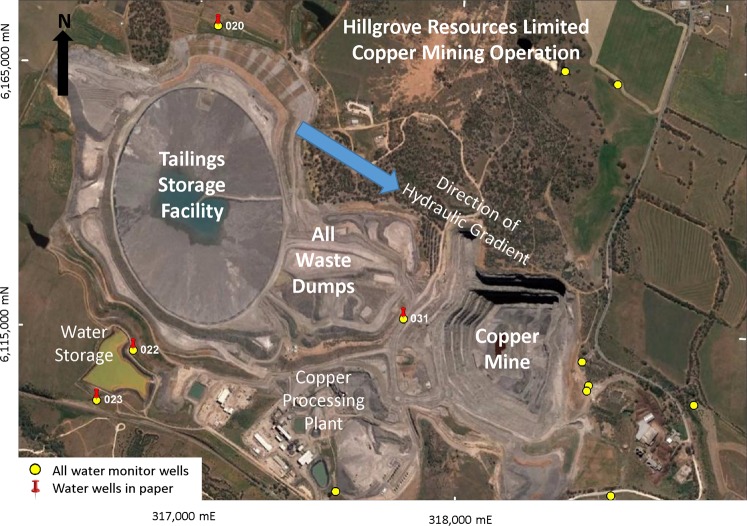
Aerial view of the Hillgrove mine site. Relevant water well sites are labelled. The integrated waste landform, comprising the waste dump and tailings storage facility, is expected on closure to be approximately 75 m above original surface. As of 2014 the foot print of the waste rock dump was approximately 70Ha, with the total footprint of the integrated waste landform being 132Ha. At this time the waste rock was assessed to contain 45.5 Mt NAF (non-acid forming) material and 48.9 Mt PAF (potentially acid forming) material. Image ©2018 Google courtesy of GoogleEarth. Image is dated 15 October 2017. Google Earth has given a general permission for its use in this manner (https://www.google.com/permissions/geoguidelines/). The figure was created using MapInfo Pro software, version 15.0 Build 18, https://www.pitneybowes.com/ie/location-intelligence/geographic-information-system/mapinfo-pro.html.

Executing the tasks to remove the risk of acid mine drainage (AMD) is the greatest environmentally-related cost for many sulfide-containing mines and construction sites around the world. Therefore characterising the AMD risk is critical to planning and remediation. Hillgrove Resources Limited is currently extracting unoxidised material from an open pit near Kanmantoo in South Australia (Fig. 1). The usual host rock in the open pit is a garnet-andalusite-biotite schist (GABS) within which are zones of copper sulfides characterised by chlorite, magnetite, quartz, chalcopyrite, pyrrhotite with minor pyrite. The principal garnet is Fe rich almandine.

The Kanmantoo open pit has been mined intermittently since 1970. In the period 1970 to 1976 the copper bearing material was mined and processed, and the non-ore material from the open pit was dumped nearby as waste rock. The waste rock dump was not capped upon mine closure in 1976 and has been open to the environment for over 40 years. There was no sulfur assaying of the rock at the time of dumping, but recent studies from the current mining operation suggest that the sulfur content of the 1970’s waste dump is between 0.1 to 5 wt%.

Hillgrove Resources Limited re-opened the open pit for mining in 2011 and has been dumping waste rock with varying sulfur content since that time over the top of the 1970’s waste rock dump. From 2011 fresh waste rock was defined as potentially acid forming (PAF) if the total sulfur content was >0.3 wt% and thus requiring, by statutory regulation, encapsulation at end of mine life to remove the risk of AMD. Waste rock with less than 0.3 wt% sulfur is dumped separately and is to be used as the encapsulation material around and over the PAF material at the closure of the open pit.

Rigorous groundwater quality monitoring is undertaken at the Kanmantoo mine site by third-party consultants. Well 020 is upstream of the tailings storage facility (TSF) and the waste dump, wells 022 and 023 are downstream of the TSF, and well 031 is downstream of the waste dump. Regional drainage is to the south-east. These wells are most likely to have been impacted by any water run-off from the waste dumps and the TSF (see Fig. 1 for well placements). Note that the run-off from the existing waste dump is a combination of Hillgrove (post-2011) and historic (1970–1976) mine waste rocks. The pH of waters from these wells has been measured since 2011 and ranges from 6.68 to 7.36 with the exception of well 031 which was initially alkaline with pH 11.7–12.38. Only well 031 was drilled with foam, causing this alkalinity, due to the broken ground conditions. The foam has since been flushed from this hole and pH is now in the range 6.68–7.36. Elevated metal concentrations have not been observed. These water well monitoring results appear to indicate that the 1970’s waste dump, whilst exposed to atmospheric oxidation for over 40 years, has not contributed to AMD. These results are not consistent with the predictions from the standard acid-base accounting (ABA) tests that characterise rocks with >0.3 wt% S as having potential to generate AMD.

A new study of the AMD potential of these rocks was initiated in 2017 to understand the apparent discrepancy between ABA testing and field observations. In particular the aim was to ascertain, by detailed examination of the acid and neutralisation generation capacities and rates, whether waste rock with greater than 0.3 wt% sulfur has the potential to generate acid. If this test work was to result in the definition of a significantly greater volume of rock as non-acid forming (NAF) than previously characterised, then there would be a significant impact on environmental planning and a great opportunity to create a substantially thicker encapsulation cover over the PAF material.

Initial examination of the waste rock in 2017 indicated that the standard internationally accepted ABA tests to identify PAF rocks may not be suitable for characterising the potential for AMD from these rocks given their documented sulfide and silicate mineralogy. This paper presents the results of a re-examination of the assumptions in the standard ABA tests and the reasons why they fail in this instance, and a description of an alternative method, using readily accessible laboratory facilities to improve the prediction of AMD for materials characterised by low-pyrite and low-carbonate content. Potentially, these revised methods have wide-ranging applications across many extraction and construction sites where characterising the AMD potential of low pyrite-containing sulfidic materials is important.

### Acid mine drainage classification

In Australia, the Environmental Protection Agency’s accepted classification of the AMD potential of a waste rock is based on the assessment of a number of rock samples of net acid producing potential (NAPP) and net acid generation pH (NAGpH)^1–3^. NAPP is equal to the maximum potential acidity (MPA) minus the acid neutralising capacity (ANC); NAPP = MPA–ANC. MPA is calculated as (total sulfur wt%) × 30.6. ANC is measured by the standardised addition of hydrochloric acid to 2 g of sample with back-titration using sodium hydroxide solution to pH 7 to determine the amount of acid consumed by reaction with the sample. The values of both MPA and ANC are commonly given in units of kg H_2_SO_4_ tonne^−1^ of rock.

NAGpH is a standard measured value for which the sample is reacted with hydrogen peroxide, to provide accelerated oxidation, leading to simultaneous acid generation and neutralisation reactions. After reaction overnight, the sample is heated to ensure the reaction of any remaining sulfides and is then left to cool before the NAGpH is measured^1^.

On the basis of NAPP and NAGpH the acid generating characteristics of mine wastes are defined using the following three categories:

PAF – potentially acid forming, NAGpH <4.5 and NAPP >0;

NAF – non-acid forming, NAGpH >4.5 and NAPP <0;

UC – uncertain, NAGpH >4.5 and NAPP >0 or NAGpH <4.5 and NAPP <0.

**Figure 2:**
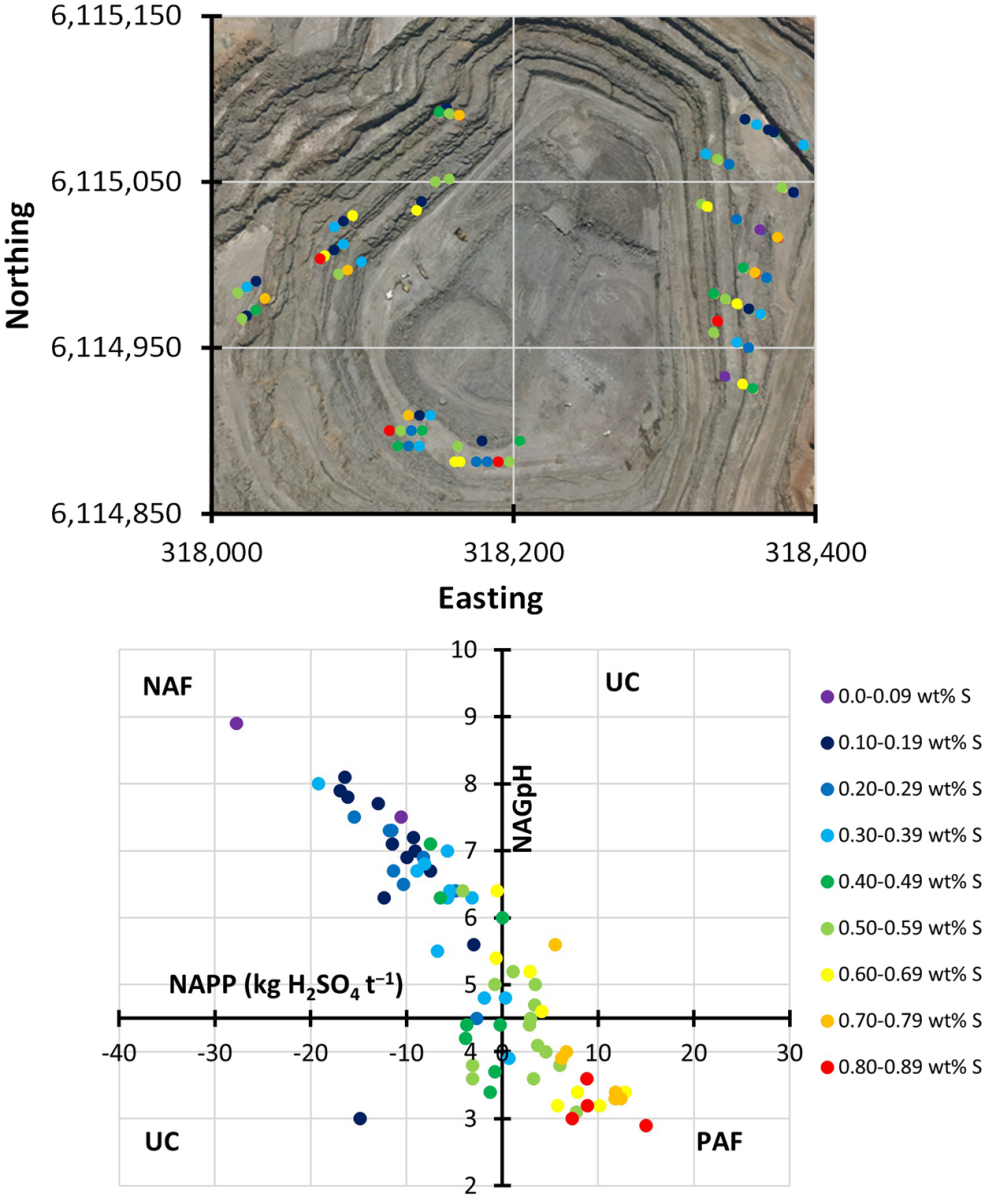
Characterisation of the 74 samples. Top – sampling locations overlaying an image of Hillgrove Resources Limited copper mine. The underlying satellite image, dated 31 Jan 2014, is owned by Hillgrove Resources Limited. Hillgrove Resources Limited gives its permission to be used in this manner for this document. The figure was created using MapInfo Pro software, version 15.0 Build 18, https://www.pitneybowes.com/ie/location-intelligence/geographic-information-system/mapinfo-pro.html. Bottom – AMD assessment using standard approaches. The legend is common to both top and bottom. Neither the sulfur content of these samples nor their acid generating characterisation was found to be location specific. Thirty five of these samples were found to have sulfur content of 0.3–0.6 wt%.

In March 2017 Hillgrove commenced a new program of AMD characterisation test work on a suite of 74 samples from fresh waste rock to characterise the material given the water well and geologic observations. Sample location and acid-generating characteristics (based on the standard ABA tests) are shown in Fig. 2. Twelve samples were chosen from the 74 samples for further test work. Table 1 presents silicate characterisation by power X-ray diffraction (XRD) of these samples (note: sulfide minerals were not detectable using laboratory XRD) and Table 2 the total sulfur contents ranging from 0.34 to 0.67 wt%.

**Table 1:** Acid neutralising characteristics of the waste rock samples.

Sample	Quartz (wt%)	Biotite (wt%)	Almandine (wt%)	Andalusite (wt%)	Chlorite (wt%)	Amorphous (wt%)	ANC (kg H_2_SO_4_ t^−1^)	RAA (kg H_2_SO_4_ t^−1^)	RAA/ANC (%)
1	44	22	12	9	4	9	11.4	4.4	39
2	56	21	6	13	4	0	13.6	4.9	36
3	52	24	11	10	2	0	19.6	3.4	18
4	61	19	12	8	2	−2	9.7	3.4	35
5	48	20	14	9	2	7	12.0	3.4	29
6	58	20	5	11	3	4	14	4.4	32
7	59	15	9	8	1	8	16.4	6.4	39
8	46	19	13	10	2	10	17.3	7.8	45
9	56	11	14	7	5	7	19	11.0	58
10	57	20	14	8	3	−1	17.2	4.4	26
11	48	24	13	11	3	1	12.5	4.9	39
12	47	25	9	10	3	6	13.9	5.4	39
Average	53	20	11	10	3	4	15	5	36
Std. dev.	6	4	3	2	1	4	3	2	10

From left to right: The composition of the samples derived from quantitative laboratory XRD analyses, acid neutralisation capacity (ANC), estimation of readily accessible alkalinity (RAA) based on acid base characterisation curve testing and the ratio of RAA/ANC.

**Table 2:** Acid generating characteristics of the waste rock samples.

Sample	Total S	NAGpH	NAPP	Asgmt. NAPP	Sulfide S	NAPP*	Asgmt. NAPP*	Py S	Pyrr S	Chalc S	NAPP**	Asgmt. NAPP**
1	0.63	3.4	7.9	PAF	0.524	4.6	PAF	0.18	0.35	0.00	−3.9	UC
2	0.42	3.7	−0.8	UC	0.326	−3.6	UC	0.10	0.23	0.00	−9.3	UC
3	0.54	3.8	−3.1	UC	0.417	−6.8	UC	0.14	0.27 (0.21)	0.00	−13.6	UC
4	0.34	3.9	0.7	PAF	0.278	−1.2	UC	0.11	0.17	0.00	−5.4	UC
5	0.54	4.0	4.5	PAF	0.432	1.2	PAF	0.11	0.32	0.01	−6.8	UC
6	0.45	4.4	−0.2	UC	0.383	−2.3	UC	0.23	0.15	0.00	−6.0	UC
7	0.67	4.6	4.1	UC	0.586	1.5	UC	0.20	0.38 (0.36)	0.00	−7.9	NAF
8	0.66	5.2	2.9	UC	0.543	−6.7	NAF	0.19	0.35	0.00	−9.4	NAF
9	0.60	5.4	−0.6	NAF	0.473	−4.5	NAF	0.31	0.16	0.00	−8.6	NAF
10	0.34	5.5	−6.8	NAF	0.274	−8.8	NAF	0.13	0.14	0.00	−12.5	NAF
11	0.41	6	0.1	UC	0.33	−2.4	NAF	0.23	0.07	0.03	−4.9	NAF
12	0.35	6.3	−3.2	NAF	0.278	−5.4	NAF	0.16	0.10	0.00	−8.4	NAF

From left to right for numerical data: sample ID, total sulfur (wt%), NAGpH (pH), net acid producing potential (kg H_2_SO_4_ t^−1^), assignment based on NAPP, sulfide S (wt%), NAPP* calculated using MPA* (kg H_2_SO_4_ t^−1^), assignment based on NAPP*, pyrite S (wt%), pyrrhotite S (wt%), chalcopyrite S (wt%), NAPP** calculated using MPA** and assignment based on NAPP**. The acid generating assignments based on NAGpH in conjunction with NAPP, NAPP* or NAPP** are potentially acid forming – PAF, uncertain – UC and non-acid forming − NAF.

### Maximum potential acidity

The standard calculation of MPA assumes that all sulfur is in pyrite. Pyrite oxidation results in 2H^+^ per sulfate formed; Eq. (1). A correction of the MPA calculation is to use the sulfide sulfur assay (by chromium reducible sulfur^4^) in the calculation of MPA*^5^, i.e. MPA* = (sulfide sulfur wt%) × 30.6. NAPP* is then equal to MPA*–ANC. This reduces the resulting NAPP* considerably so that, whereas previously three of the 12 samples were classified as PAF, six as UC and three as NAF, using NAPP* two, five and five samples respectively would have these classifications (Table 2).

1 $${{\rm{FeS}}}_{2}+3.5{{\rm{H}}}_{2}{\rm{O}}+3.25{{\rm{O}}}_{2}\to {\rm{Fe}}{({\rm{OH}})}_{3}+2{{\rm{SO}}}_{4}^{2-}+4{{\rm{H}}}^{+}$$

This improved estimate of NAPP* still does not take into account the variation in acid production between different sulfide containing minerals. It is known that the oxidation of pyrrhotite (Fe_(1−*x*)_S, *x* = 0–0.2) results in preferential formation of elemental sulfur rather than sulfate^6,7^ and consequently does not result in as much acid generation as predicted by MPA*. Synthetic wastes containing 5 wt% pyrrhotite together with silicates and quartz have been found to produce 6% of the expected acidity based on standard ABA during 120 weeks of standard kinetic column leaching^8^. Nickel mine tailings containing nearly 30 wt% pyrrhotite produced <1% of the expected acidity after 107 weeks of leaching^9^. In both cases, sulfur speciation analysis of the leached solids and drainage demonstrated that around 85% of the oxidised sulfur converted to elemental sulfur, which does not result in acidity, Eq. (2). The sulfate producing pathway, Eq. (3), with production of 2H^+^ per sulfate, occurs rapidly under highly oxidising conditions as experienced during the NAG test^10^ or slowly during long-term waste weathering.

2 $${{\rm{Fe}}}_{7}{{\rm{S}}}_{8}+5.25{{\rm{O}}}_{2}+10.5{{\rm{H}}}_{2}{\rm{O}}\to 7{\rm{Fe}}{({\rm{OH}})}_{3}+8{{\rm{S}}}^{0}$$

3 $${{\rm{Fe}}}_{7}{{\rm{S}}}_{8}+17.25{{\rm{O}}}_{2}+18.5{{\rm{H}}}_{2}{\rm{O}}\to 7{\rm{Fe}}{({\rm{OH}})}_{3}+16{{\rm{H}}}^{+}+8{{\rm{SO}}}_{4}^{2-}$$

The same correction may also be applied to chalcopyrite since under normal atmospheric oxidation the major sulfur-containing product is elemental sulfur with no acid generated. Under highly oxidising conditions or slowly during long-term weathering, both acid and sulfate may be produced^10,11^. Since, the quantity of chalcopyrite in these wastes is very small (chalcopyrite S wt% is given in Table 2) this potential source of acidity is not further considered.

A detailed quantitative understanding of the mineralogy is essential to make corrections to the calculation of MPA* where pyrite is not the principal sulfide. This is not straightforward for rocks with a low sulfur content and alternate methods to conventional laboratory X-ray diffraction (XRD) must be utilised. Mineral liberation analysis (MLA), is a common analytical process used to quantify the proportions of different minerals. In this study an innovative application of MLA was developed to reduce the analytical time required to quantify each sulfide mineral. The revised MLA approach, focused solely on the sulfide mineralogy, resulted in an estimate of the weight ratios of these minerals rather than absolute quantification. The relative wt% of each sulfide mineral was then apportioned pro-rata to the total wt% of sulfide sulfur in each sample. The resulting sulfide sulfur in each mineral type is given in Table 2. Of the twelve samples analysed, eleven contained <0.30 wt% pyritic sulfur and the 12^th^ sample contained 0.31 wt% pyritic sulfur. The average pyritic sulfur content was determined to be 0.17 ± 0.06 wt% and the average pyrrhotite sulfur was 0.22 ± 0.10 wt%.

Confirmation of the MLA results was obtained using synchrotron X-ray diffraction data (Supplementary Section 1) on three samples. The synchrotron data enabled definitive identification of both pyrite and pyrrhotite. Semi-quantitative analysis of pyrrhotite was possible on two of these samples. The resulting values, given as pyrrhotite sulfur wt% shown in brackets in Table 2 are in excellent agreement with the values obtained using the combined sulfide sulfur and MLA measurements providing confidence in the MLA data.

NAPP** was then calculated using MPA** of ((pyritic sulfur wt%) + (0.2 × pyrrhotite sulfur wt%)) × 30.6. The factor of 0.2 was adopted as a conservative estimate for potential acid generation capacity of pyrrhotite. All NAPP** values were negative (Table 2). This resulted in six samples being classified as NAF with the other six being UC.

### Acid neutralisation and production rates

Whilst the quantification of MPA** and ANC is required for estimating NAPP**, these values do not provide information on the rates of acid generation or neutralisation that determine the development of AMD.

Quantitative analysis by laboratory XRD (Table 1) yielded the following average silicate composition (wt%) across the twelve samples: quartz 53 ± 6, biotite 20 ± 4, almandine 11 ± 3, andalusite 10 ± 2 and chlorite 4 ± 4. The neutralisation potential of various silicate minerals has been previously studied and affirmed^12,13^. In this study, only neutralisation from almandine, Eq. (4), is considered because its dissolution rate is at least two orders of magnitude greater than chlorite or biotite, both of which are many orders greater than either quartz or andalusite^14,15^. Furthermore, the average concentration of the total carbon in the samples is 0.05 wt% and therefore carbonate minerals are not considered to be present in appreciable quantities. The neutralisation capacity of either Al or Fe from almandine is also considered to be negligible because both Al and Fe are fully hydroxylated at pH 7 or greater.

4 $$\begin{array}{c}{({\rm{F}}{\rm{e}},{\rm{M}}{\rm{g}})}_{3}{\rm{A}}{{\rm{l}}}_{2}{\rm{S}}{{\rm{i}}}_{3}{{\rm{O}}}_{12}+3{{\rm{H}}}^{+}+9.75{{\rm{H}}}_{2}{\rm{O}}+0.375{{\rm{O}}}_{2}\to \\ \,1.5{\rm{F}}{\rm{e}}{({\rm{O}}{\rm{H}})}_{3}+1.5{\rm{M}}{{\rm{g}}}^{2+}+2{\rm{A}}{\rm{l}}{({\rm{O}}{\rm{H}})}_{3}+3{{\rm{H}}}_{4}{\rm{S}}{\rm{i}}{{\rm{O}}}_{4}\end{array}$$

**Figure 3:**
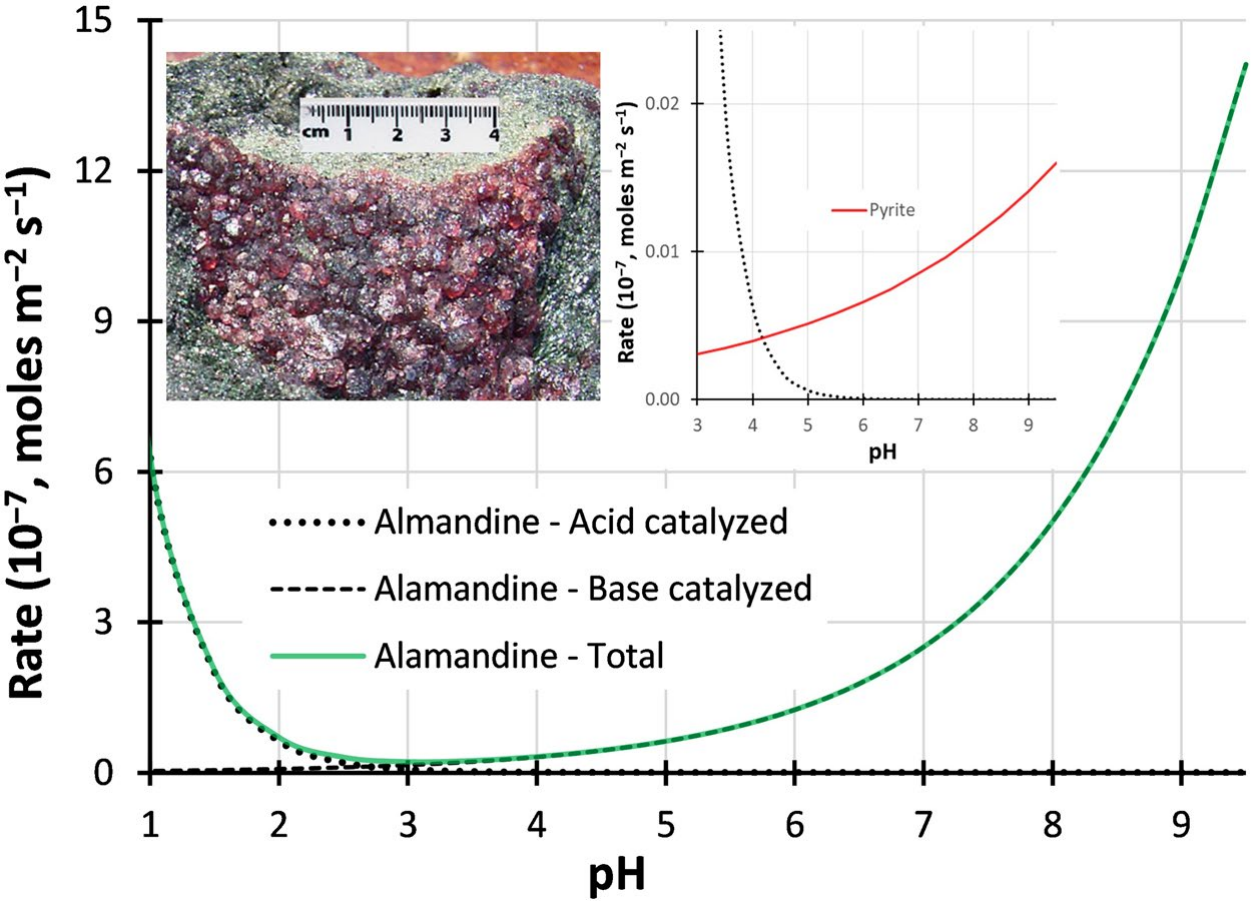
Rates of dissolution of almandine and pyrite. The main chart shows calculated rates of dissolution of almandine using both acid and base-catalysed rate laws^14^. Left inset – example of almandine waste rock. Right inset – rates of dissolution of acid-catalysed almandine dissolution compared to pyrite dissolution (O_2_ oxidation only^16^).

For almandine, both acid- and base-catalyed dissolution reactions exist, both leading to neutralisation, and therefore it is the sum of these rates that should be considered. The rate of neutralisation, calculated using Eq. (5) and the rate constants (*n*_acid_, *n*_base_, ${K}_{{\rm{acid}}}^{298.15{\rm{K}}},{K}_{{\rm{base}}}^{298.15{\rm{K}}}$) given for almandine^14^, decreases with increasing pH until approximately pH 3 and then increases again. (Fig. 3). Identification of this behavior is crucial to understanding the AMD behavior of this system.

5 $${\rm{log}}({\rm{r}}{\rm{a}}{\rm{t}}{\rm{e}})=\,({\rm{log}}\,{({\rm{K}}}_{{\rm{a}}{\rm{c}}{\rm{i}}{\rm{d}}}^{298.15{\rm{K}}})-{{\rm{n}}}_{{\rm{a}}{\rm{c}}{\rm{i}}{\rm{d}}}{\rm{p}}{\rm{H}})+\,({\rm{log}}\,{({\rm{K}}}_{{\rm{b}}{\rm{a}}{\rm{s}}{\rm{e}}}^{298.15{\rm{K}}})-{{\rm{n}}}_{{\rm{b}}{\rm{a}}{\rm{s}}{\rm{e}}}{\rm{p}}{\rm{H}})$$

The rate law, Eq. (6)^16^, for pyrite assumes dissolution driven by oxidation due to O_2_ rather than Fe^3+^, which will only occur at low pH where Fe-oxyhydroxides do not precipitate from leachates. For Eq. (6), the dissolved O_2_ (DO) is assumed to be 8 ppm (0.0005 M) equating to fully air saturated water.

6 $$rate={10}^{-8.19}{[DO]}^{0.5}/{[{H}^{+}]}^{0.11}$$

Stirred dissolution ensures that Eqs (5) and (6) represent the maximum possible dissolution rates, unlike most AMD environments, where bulk diffusion is the usual process of dissolution. Nevertheless they remain valid for comparative and interpretive purposes.

The right insert in Fig. 3 presents a common error made when considering neutralisation capacity where the rate of acid-catalysed almandine neutralisation is compared to the rate of pyrite dissolution, and the contribution of base-catalysed almandine neutralisation is not considered. Consideration of this graph in isolation is likely to lead to the conclusion that there is an insufficient rate of almandine dissolution to provide effective neutralisation of the acid released due to pyrite dissolution.

However, critically, it is not solely the rate of dissolution per mole of the various minerals that is important because the mass (mol) fraction of the minerals must also be accounted. That is, the absolute or ‘gross’ rates of production of acid or neutralisation must be considered to fully assess the possibility of AMD. The following discussion will describe a process to incorporate the relative proportions of the sulfide and silicate minerals into acid neutralisation rate (ANR) and acid generation rate (AGR) calculations using the process described below, to interpret standard AMD characterisation measurements.

### Acid Neutralisation Rate (ANR)

1. The mass fraction of the given mineral is converted into moles mineral per kilogram (*M* mol kg^−1^).
2. The *rate* of mineral dissolution (mol m^−2^ s^−1^) for the desired pH is calculated using Eq. (5) to obtain a total rate of dissolution.
3. The neutralisation capacity, *NC*, for one mole of almandine is 3; Eq. (4). The rate of neutralisation (mol m^−2^ s^−1^) is then calculated by *M* × *rate* × *NC*.
4. ANR (mg H_2_SO_4_ kg^−1^ week^−1^) is then calculated assuming a surface area of 1 m^2^ and applying the conversion factor (98/2) × 1000 × 7 × 24 × 36000. To convert to kg H_2_SO_4_ t^−1^ week^−1^ divide by 1000.

### Acid Generation Rate (AGR)

In this calculation we consider that 20% of the pyrrhotite reacts in the same manner and at the same rate as pyrite.

1. The effective pyrite mass fraction is calculated as 119.98 × (pyrite S wt% + (0.2 × pyrrhotite S wt%))/(2 × 32.065 × 100). The mass fraction is then converted into moles pyrite per kilogram (*M* mol kg^−1^).
2. The *rate* of (effective) pyrite dissolution (mol m^−2^ s^−1^) for the desired pH is calculated using Eq. (6).
3. 2H^+^ are formed per sulfate, i.e., acidification capacity (*AC*) = 2. The acid generation rate (mol m^−2^ s^−1^) is then calculated by *M* × *rate* × *AC* and the same calculation as for step (4) above gives AGR in kg H_2_SO_4_ t^−1^ week^−1^.

With extended oxidation times, the elemental sulfur generated by the oxidation of the pyrrhotite (Eq. (2)) will eventually fully oxidise through to sulfate which is an acid generating reaction; Eq. (7).

7 $${{\rm{S}}}^{0}+{{\rm{H}}}_{2}{\rm{O}}+\frac{3}{2}{{\rm{O}}}_{2}\to {\rm{S}}{{\rm{O}}}_{4}^{2-}+2{{\rm{H}}}^{+}$$

A study of the rate of this reaction in relation to storage of elemental sulfur has been carried out under relatively comparable conditions^17^ to this study. Nutrient poor oxidation, as likely in a waste rock dump, i.e. with limited contribution from microbially catalysed oxidation, resulted in oxidation rates of 0.06–0.08 μg S^0^ cm^−2^ day^−1^ across 12–32 °C. Using the following assumptions (1) a specific surface area of 1 m^2^ g^−1^, (2) the complete conversion of 0.8 of the sulfur within the pyrrhotite to elemental sulfur and (3) a rate of 0.07 μg S^0^ cm^−2^ day^−1^ an AGR of 2.06 × 10^−3^ kg H_2_SO_4_ t^−1^ week^−1^ is calculated. This AGR will be overestimated because the concentration of elemental sulfur applied is greater than that realistically present at any one time. The relationship between this rate and the rate of acid generated by pyrite and 0.2 of the pyrrhotite will be discussed further.

### Acid Neutralisation Capacity (ANC)

In the calculation of NAPP (and variants) the ANC is subtracted from the maximum potential acidity (MPA). ANC, given in Table 1 for the twelve samples, is measured via titration^1,18,9^ and it is therefore useful to examine the assumptions inherent in the chemistry of this titration test work. It is broadly understood that this approach enables quantification of neutralisation primarily from carbonate minerals^2^.

For the standard ANC titration an initial slurry pH of 0.8 is estimated, prior to back-titration to pH 7. At pH 0.8, the average ANR (using the same speciation as for pH 7) is calculated, using 11 wt% almandine, to be 2.3 × 10^2^ kg H_2_SO_4_ t^−1^ week^−1^ but this rate rapidly drops as the pH increases. For instance, at pH 1.5 the rate is approximately 20% of that at pH 0.8. These calculations assume full hydrolysis of Fe and Al, as per Eq. (4), so that the dissolution of these species does not contribute to the calculated ANR. In comparison to calcite, a commonly cited source of neutralisation, this rate is slow. For the same average moles per kg as almandine, calcite would release neutralisation at >10^6^ kg H_2_SO_4_ t^−1^ week^−1^ at pH 0.8 (using kinetic data from^14^).

The total ANC calculated at pH 7 (as per Eq. (4)) to be available on average from the almandine mass fraction is 38 kg H_2_SO_4_ t^−1^ as compared to the average measured ANC value of 15 ± 3 kg H_2_SO_4_ t^−1^. It is therefore apparent that the measured ANC only accesses approximately 40% of the neutralisation capacity available from almandine, without considering the additional neutralisation capacity of chlorite or biotite. This is due to the slower kinetics of almandine dissolution as compared to carbonate dissolution, for which this test is designed. However, inclusion of the calculated ANC in the NAPP** calculation would not change the AMD classification of six of the twelve samples from UC to NAF because the UC classification is due to the NAGpH of these samples being less than 4.5.

It should be noted that MPA* is equal to the maximum possible acidity assuming that all elemental sulfur formed is eventually fully oxidised through to sulfate. The average MPA* was calculated to be 12 ± 3 kg H_2_SO_4_ t^−1^, approximately one third of the total ANC calculated to be due to almandine (assuming full hydrolysis of Fe^3+^ and Al^3+^). The maximum AGR at pH 7 is calculated to be 0.019 kg H_2_SO_4_ t^−1^ week^−1^ (using wt% of pyrite sulfur + 0.2×pyrrhotite sulfur acting as pyrite + 0.8 × pyrrhotite sulfur acting as elemental sulfur) compared to ANR at pH 7 of 57.2 kg H_2_SO_4_ t^−1^ week^−1^. Given these figures the possibility of AMD, even taking into account potential acid generation due to the presence of elemental sulfur, would appear remote.

### Net Acid Generation (NAG)

The standard accelerated oxidation NAG test uses 15 vol.% peroxide, which oxidises all sulfides completely through to sulfate and acid. This results in an overestimation of the actual acid produced where pyrrhotite, or other low-acid producing sulfides, are a significant proportion of the sulfides present. Acid generation and neutralisation reactions occur simultaneously during the NAG test; however, the actual extent of neutralisation will be pH dependent. It is therefore difficult to apply improvements to the values resulting from the NAG test even when the mineralogy is known. For the Kanmantoo samples, the values of titratable acidity of the resulting NAG slurry to both pH 4.5 and 7.0 were very low at ≤4.8 kg H_2_SO_4_ t^−1^ (for the 6 samples with NAGpH <4.5) and ≤9.2 kg H_2_SO_4_ t^−1^ respectively, even with full reaction of the sulfide minerals via acid producing mechanisms. Hence, the assignment of six samples as UC on the basis of NAGpH (Table 2) is likely to be an artefact of the highly oxidising, and unnatural, nature of the NAG measurement.

### Paste pH

Standard paste pH measurements were carried out by contacting 25 g of each sample with 50 ml of deionised water overnight after which the slurries had pH of 9.1–9.8. Standard NAPP analysis (Table 2) would suggest that at least some of these measurements should be acidic. The calculated average AGR at pH 5.5, the approximate starting pH of the test, taking into account total pyrite and 0.2 of the pyrrhotite present is calculated to be 0.012 kg H_2_SO_4_ t^−1^ week^−1^ as compared to the calculated ANR of 20.3 kg H_2_SO_4_ t^−1^ week^−1^ with the main contribution to the ANR being from the base-catalysed dissolution of almandine. This AGR value is approximately 6 times greater than the rate of acid generation from the possible presence of elemental sulfur (2.06 × 10^−3^ kg H_2_SO_4_ t^−1^ week^−1^), calculated assuming unrealistically that 80% of the pyrrhotite sulfur is present as elemental sulfur. Even taking into account the contribution to AGR by elemental sulfur the greater rate of acid neutralisation compared to acid production explains why, in reality, these slurries are alkaline rather than acidic.

### Acid buffering characteristic curves (ABCC)

Acid buffering characteristic curves (ABCC) are generally used to assess whether neutralisation capacity is readily available. This standard test involves a slow titration of the sample with acid while monitoring pH^1^. Readily accessible alkalinity (RAA) is taken to equal the amount of acid addition required to reach pH 4.5 during the ABCC test and is shown in Table 1 for the twelve samples. During this titration it is likely that some proportion of Al^3+^, Fe^2+^ and Fe^3+^ are dissolved to solution. Incomplete oxidation of Fe^2+^ and/or incomplete hydrolysis of Fe^3+^ and Al^3+^ would result in apparent acid consumption whereas given a longer-term delay and complete reaction the total of these processes, i.e. dissolution, oxidation and hydrolysis, will neither consume nor produce acid. An incomplete reaction would result in overestimation of the RAA. However, the rapid pH decrease and lack of appreciable pH plateaus in the ABCC curves (Supplementary Section 2) suggest that there is little acid buffering and that incomplete hydrolysis is not an appreciable component of the RAA with small values across 3.4–11.0 kg H_2_SO_4_ t^−1^. The % ratio of RAA as compared to the measured ANC is on average 36 ± 10.

To fully appreciate this result, the AGR in the ABCC test (i.e. the rate of acid addition) is compared to the calculated ANR for a range of pH. The acid addition of 0.2 ml of 0.1 M HCl every 1000 seconds for 2 g of solids equates to an AGR of 2.97 × 10^5^ kg H_2_SO_4_ t^−1^ week^−1^. The ANR for the average almandine content of these twelve samples at pH 7 is 57.2 kg H_2_SO_4_ t^−1^ week^−1^ and at pH 4.5 is 10.2 kg H_2_SO_4_ t^−1^ week^−1^. Clearly the rate of neutralisation released by the almandine during the ABCC test cannot match the rate of acid addition and therefore the pH will rapidly decrease.

The AGRs from the average pyrite and pyrrhotite content of these twelve samples at pH 7 and 4.5 are calculated to be 0.0168 and 0.009 kg H_2_SO_4_ t^−1^ week^−1^ respectively. Even taking into account the possibility of long term elemental sulfur oxidation and the consequent formation of acid, which at most is calculated to be 2.1 × 10^−3^ kg H_2_SO_4_ t^−1^ week^−1^, the rate of supply of neutralisation, due to the greater natural abundance of almandine, will more than compensate for the acid generation. While the accessible neutralisation appears low, its rate of release is considerably greater than the rate of release of acid from pyrite and pyrrhotite when the relative amounts of these minerals are taken into account. It is for this reason that these waste rocks will not be acid producing and readily explains the neutral pH water well measurements from 40 year old waste dumps.

### Implications for prediction of acid mine drainage

The standard methods for predicting AMD are designed for mineral systems where the dominant sulfide mineralogy is pyrite and where neutralisation is dominated by carbonates as calcite or dolomite. However, in the presence of sulfide minerals other than pyrite, the NAPP results and the highly oxidative NAG measurement can be misleading. In addition, the ANC and ABCC measurements only provide an assessment of rapidly dissolving sources of neutralisation (for example carbonates) and will not capture most silicate-based neutralisation. The combined effect of these standard ABA test on non-pyrite and non-carbonate mineralogy may result in an erroneous assessment of AMD. In these instances, NAPP** can be estimated from the sulfide and neutralising minerals present in the rocks based on known dissolution mechanisms and mineralogy. For an alternative process of assessing AMD as described herein, the mineralogy of the rocks and associated sulfides is undertaken by laboratory XRD, and supplemented by sulfide sulfur assay. In the event that the sulfide concentration is too low for XRD to provide useful quantification, MLA (or QEM-SCAN, quantitative evaluation of materials by scanning electron microscopy) analysis can be utilised. A more complete assessment of the relative rates of ANR and AGR can then be calculated using the quantified mineralogy with known mechanisms and rate laws.

In the Hillgrove example, after external peer review conducted by the Department of Premier and Cabinet (South Australia) this analytical approach resulted in a reclassification of the AMD potential of waste rock with less than 0.6 wt% total sulfur with considerable benefit for long-term dump remediation. Ongoing monitoring of water well pH is continuing and further verification of these results will be undertaken as more data is available.

## Method

### Sampling

In February 2017 a suite of 74 samples were collected from various areas within the open pit of the copper mining operation for AMD test work.

The samples were from production drill holes collected as follows. A percussion drill rig drills a 140 mm diameter hole vertically into the floor of the open pit. The cuttings generated by the percussion drill bit are blown by the compressed air back up the drill hole to the surface and into a sample collection boot at the collar of the hole. The sample material in the sample collection boot is sucked through a large diameter pipe to a collection cyclone, where the material is dumped into a storage container. Every 6 metres the material collected by the storage container is dumped through a riffle splitter and split down to <2 kg. The entire 2 kg sample is pulverised to <75 μm and a 1 g aliquot taken for acid digest.

The samples collected contained total sulfur between 0.08 wt% and 0.89 wt% and were hosted in garnet-andalusite-biotite schist.

### Standard Acid Mine Drainage Testing

The standard methods of testing of acid neutralisation capacity (ANC), net acid generation (NAG), paste pH and acid base characterisation curves are provided in Miller *et al*.^1^. Here we only provide further information relevant to how these tests were conducted.

ANC – The concentration and volume of acid to be added is determined by the “fizz rating”, the measurement of which is described in Miller *et al*.^1^. The fizz rating was determined to be 1 in all cases. The test then entails the addition of 8 mls of 0.5 M HCl and 20 ml of water to 2 g of pulverized sample. This total solution of 28 mls would contain 0.14 M of HCl, equating to a starting pH of approximately 0.8.

NAG – Single addition NAG testing was undertaken. Sequential NAG testing was unnecessary due to the small sulfide content of the samples resulting in full dissolution during a single NAG stage.

### Mineral Liberation Analysis

MLA was performed on polished 1″ mounts coated with 20 nm of carbon using a tungsten source FEI MLA650 scanning electron microscope (SEM) located at the Central Science Laboratory, University of Tasmania. It is equipped with a Bruker Quantax Esprit 1.9.4 energy dispersive X-ray spectrometry (EDS) system with two XFlash 5030 SDD detectors with 133 eV energy resolution.

An accelerating voltage of 20 kV and beam current of around 7 nA were used, yielding a total EDS X-ray intensity of around 600,000 cps and dead time of 30% on quartz. Backscattered electron (BSE) imaging contrast and brightness were adjusted to separate sulfide grains from most of the silicates present.

MLA software version 3.1.5 and the method SPL_XBSE were used. The method is optimized for finding accessory minerals in polished rock samples. SPL_XBSE settings were 1000 × 1000 pixels resolution, 1 mm field of view, and 8 s acquisition time per frame (corresponding to a pixel size of 1 × 1 µm^2^). The spectrum acquisition time was 5 ms for mineral grains at least 2 pixel in size. An area of 23 mm diameter was scanned on each mount (≈437 frames).

Only the grey-scale of the backscattered electron images that includes the sulfide minerals was measured. Each particle within this window was then examined in turn and an energy dispersive spectral map for each particle was generated. Generally a matching threshold of database to measured spectra of 70% is used for identification. However, in this case it was increased to 95% to differentiate pyrite from pyrrhotite as their spectra are similar. This has the side-effect of discriminating against smaller particles where neighbouring particles or the resin matrix may have some influence on the spectra recorded. So the assumption inherent in the analysis is that the sulfide minerals fragment in the same way on grinding and no discrimination is introduced.

### Laboratory X-ray Powder Diffraction

XRD data collection of the twelve waste samples were conducted on a Bruker D4 Endeavor diffractometer with Co Kα (1.7902 Å) radiation at 30 kV and 20 mA. Diffraction patterns were collected with θ/2θ geometry from 5 to 80 °2θ at 0.02 °2θ increments with a rotating sample stage. All the samples were ground to less than 38 μm. Corundum (α-Al_2_O_3_, 15 wt%) was added as an internal standard.

Phase identification was carried out using the DiffracPlus EVA software (Bruker) with the ICDD-PDF2 database (International Center for Diffraction Data, 2000). Bruker-AXS TOPAS V4.2 software was phase quantification.

### Synchrotron X-ray Powder Diffraction

Three samples were analysed at the ANKA synchrotron (Karlsruhe Institute of Technology) powder diffraction beamline. All samples had previously been micronised and mixed with 15 wt% corundum.

Data from sample 7 (containing 0.586 wt% sulfide, UC) was collected in steps of 0.01° 2θ from 5 to 60 °2θ. Data from samples 1 and 3 were collected in two stages: 5–30°2θ with step size 0.006 °2θ and 30–55 °2θ with step size 0.01 °2θ.

The incident X-ray wavelength was 0.8856 Å. The 2θ range 5–55° corresponds to approximately 9–106° for Cu Kα radiation. All data was normalised relative to the average X-ray beam intensity as measured by a reference ion chamber.

## Supplementary information

Unexpected Non-acid Drainage from Sulfidic Rock Waste
