# Supplementary material for: Unexpected Non-acid Drainage from Sulfidic Rock Waste
Source: Sci Rep. 2019 Mar 13;9:4357. doi: 10.1038/s41598-019-40357-4 (PMC6416257; doi:10.1038/s41598-019-40357-4)
Supplement: Supplementary file 1 — Unexpected Non-acid Drainage from Sulfidic Rock Waste [file 41598_2019_40357_MOESM1_ESM.pdf]

Unexpected Non-acid Drainage from Sulfidic Rock Waste

Andrea R. Gerson[1]*, Peter J. Rolley[2], Catherine Davis[2], Sandrin T. Feig[3], Stephen Doyle[4], Roger St.C. Smart[1]

[1] Blue Minerals Consultancy, Wattle Grove, Tasmania, Australia, [2] Kanmantoo Copper Mine, Éclair Mine Road, Kanmantoo South Australia, [3] Central Science Laboratory, University of Tasmania, Hobart, Tasmania, Australia, [4] Institute for Photon Science and Synchrotron Radiation (IPS), Karlsruhe Institute of Technology (KIT), Hermann-von-Helmholtz-Platz 1, 76344 Eggenstein-Leopoldshafen, Germany
* e-mail: Andrea@bluemineralsconsultancy.com.au

S1. Synchrotron X-ray Powder Diffraction

The resulting diffraction patterns are shown in three images (Supplementary Figure 2) of decreasing 2θ range. Pyrite and pyrrhotite diffraction peaks become apparent in the bottom image of greatest resolution. Note that these peak heights are less than approximately 60 counts as compared to approximately a peak height of 20,000 for quartz in the top diffraction image and commonly around 2,000 for other phases.

A full quantitative phase analysis was not carried out as the objective of these measurements was to identify and quantify, if possible, relative amounts of sulfide containing phases. In the bottom image of Figure 1 two isolated pyrrhotite diffraction peaks are indicated at 17.1 and 24.7 °2θ with a shoulder at approximately 19.2 °2θ. For pyrite two very small peaks, one isolated peak and a shoulder are indicated at approximately 16.3, 23.1, 18.8 and 21.1 °2θ, respectively.

Semi-quantitative analysis was carried out by fitting the corundum diffraction peaks at 20.0, 21.5 and 24.5 °2θ and the two isolated pyrrhotite diffraction peaks. The quantitative refinement of pyrrhotite concentration using the two isolated peaks was stable for samples 3 and 7. It was not possible to fit pyrite as the refinement was not stable, and therefore not reliable, because there was only one significant isolated peak. As the wt% of corundum is known it is possible to use this to calculate the absolute wt% of pyrrhotite, and therefore wt% sulfide due to pyrrhotite, in the samples (with corundum normalised out). This can then be compared to the CRS wt% sulfide.

Sample 3 = 0.58 wt% pyrrhotite
= 0.21 wt% pyrrhotite sulfur

Sample 7 = 0.98 wt% pyrrhotite
= 0.36 wt% pyrrhotite sulfur

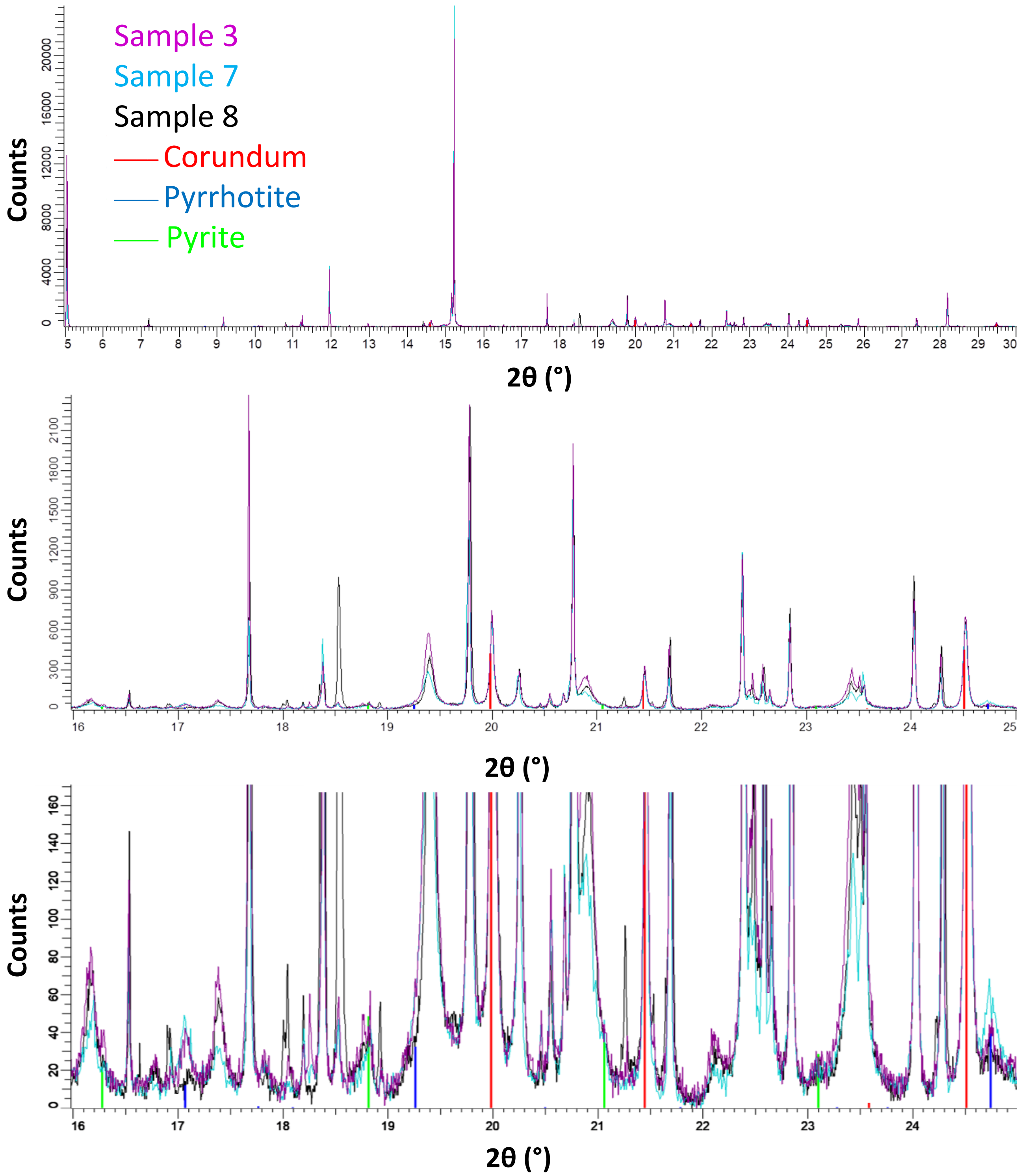

Supplementary Figure 1 Synchrotron diffraction patterns of samples 3, 7 and 8. Diffraction peak locations for corundum, pyrrhotite and pyrite are shown by vertical red, blue and green lines respectively.

S2. Acid buffering characteristic curves

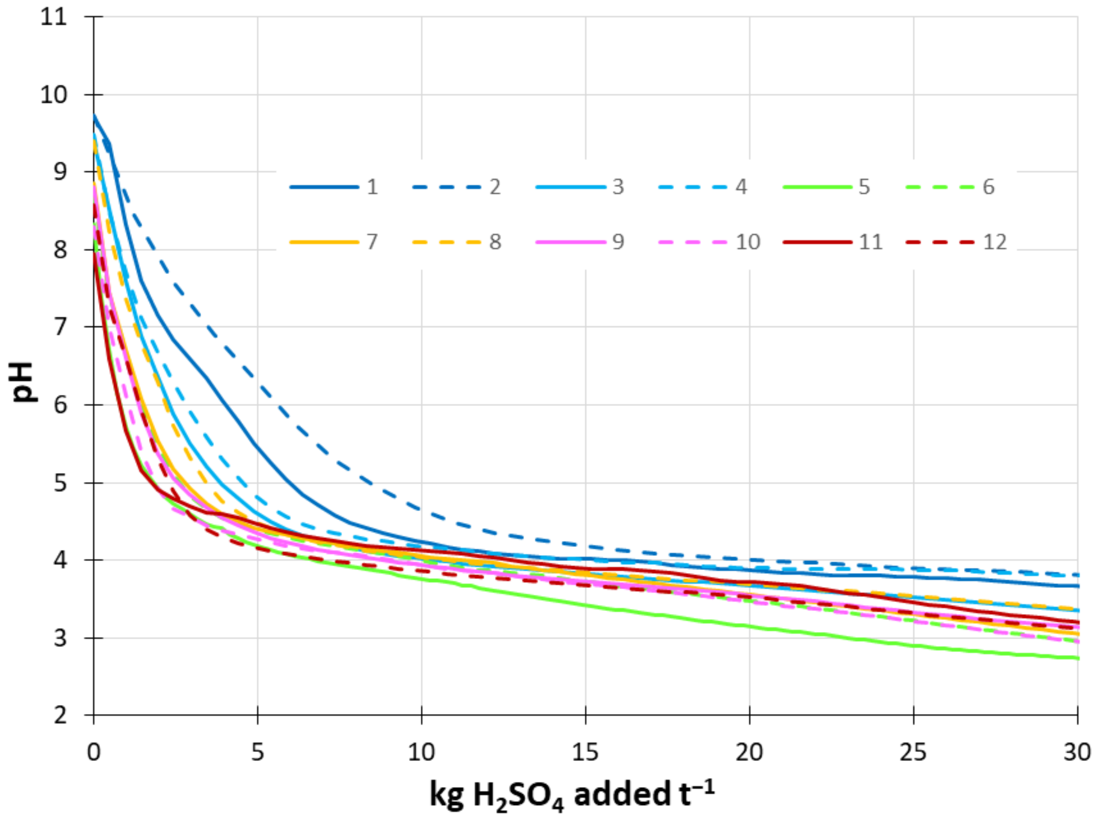

Supplementary Figure 2 Acid base buffering characteristic curves for the 12 samples.
